# Bleeding Gastrointestinal Stromal Tumor (GIST) Results in a Sticky Situation: The Good and Bad of Hemostatic Spray

**DOI:** 10.7759/cureus.46691

**Published:** 2023-10-08

**Authors:** Azfar S Syed, Amberly R Vaughan, John G McCarthy, Jeffrey T Laczek

**Affiliations:** 1 Gastroenterology, Walter Reed National Military Medical Center, Bethesda, USA; 2 Internal Medicine, Keesler Medical Center, Biloxi, USA

**Keywords:** imatinib therapy, bleeding gist, treatment complication, endoscopic hemostasis, upper endoscopy

## Abstract

A 50-year-old female presented with symptomatic anemia and hematemesis due to a 3.3 cm gastric gastrointestinal stromal tumor (GIST), which was located in the fundus. Adequate endoscopic views were only achieved in the retroflexed position and attempts at hemostasis via endoscopic clips were unsuccessful. Subsequently, TC-325 hemostatic powder was sprayed on the bleeding lesion and given retroflexed positioning, the powder also coated the esophagogastroduodenoscopy (EGD) scope where it abutted the gastroesophageal junction (GEJ). Hemostasis was successful, but the scope was unable to be withdrawn due to adherence to the surrounding mucosa. With torque maneuvering and a moderate amount of withdrawal force, the scope was successfully freed. The patient was started on imatinib mesylate and did not experience further bleeding episodes. This case highlights the challenge of achieving hemostasis in a bleeding GIST, the beneficial role of hemostatic powder spray, and the need for caution when utilizing it in a retroflexed manner.

## Introduction

Gastrointestinal stromal tumors (GISTs) are neoplasms that arise from the muscular wall of the gastrointestinal tract and can cause gastrointestinal bleeding. Although rare, GISTs are the most common mesenchymal tumor of the gastrointestinal tract. They tend to arise from the interstitial cells of cajal which are the pacemaker cells of the gastrointestinal tract [1]. The incidence of GISTs in the United States is approximately 0.68-0.78/100,000 [2]. Males and females are almost equally affected, and patients are most commonly diagnosed in the seventh decade of life [3]. The diagnosis is based on histology, and immunohistochemical staining for CD117, DOG1, and CD34 [4].

Common initial presentations for patients with GIST include gastrointestinal bleeding, abdominal pain, and early satiety, although discovery may also be incidental [5]. Bleeding is due to tumor growth resulting in tissue necrosis due to decreased blood flow; albeit the exact mechanism is not known [6]. Other causes of bleeding include the overall vascular nature of this tumor and direct invasion into blood vessels. Bleeding GISTs tend to have a worse overall survival rate, but the overall recurrence-free survival is the same. Bleeding is more likely to occur in larger tumors with higher mitotic rates and in the small intestine [7].

A mutation in the KIT proto-oncogene is thought to be the driving factor for the tumor growth and imatinib mesylate, which is a tyrosine kinase inhibitor, has a well-established role in the treatment of GIST by reducing its size and potential for recurrence. Nonetheless, there is a paucity of data on its benefit in treating bleeding GIST [4].

Radiation therapy is another treatment modality in malignancies that currently has no established role in the management of GISTs outside of palliation in bone metastases. However, these tumors may be radiosensitive, and more data are needed to determine a role for radiation treatment [8,9]. Comparatively, hemostatic powder is a relatively new modality to control gastrointestinal hemorrhage. It has the benefit of not requiring exact precision to be effective. This case highlights the utility of hemostatic powder spray, the potential complications of applying hemostatic powder spray, and the potential benefit of imatinib to treat gastrointestinal hemorrhage related to a GIST.

## Case presentation

**Figure 1:**
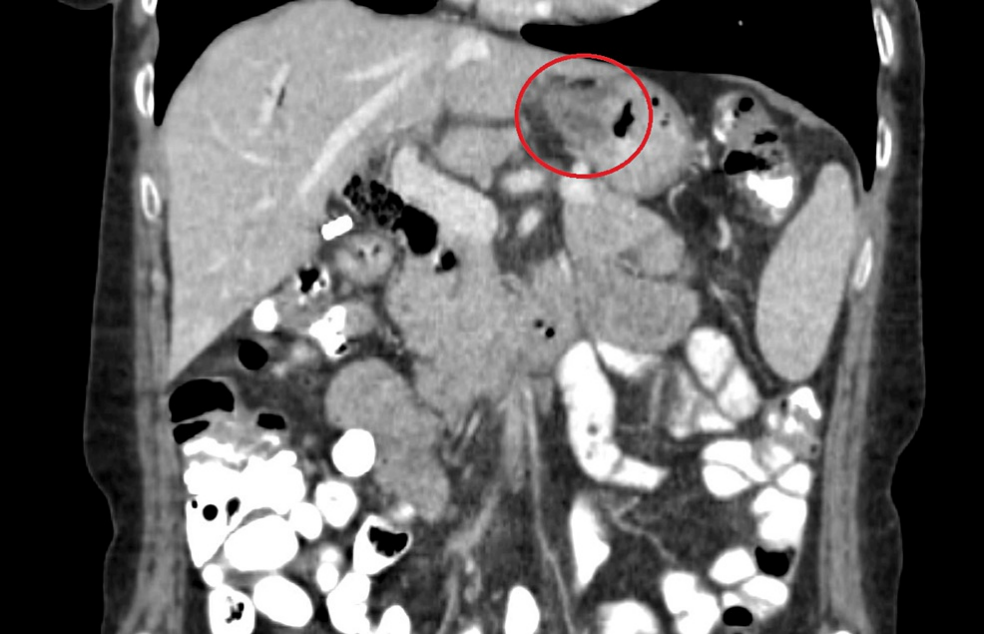
CT scan of the gastric mass

A 50-year-old female with rheumatoid arthritis and no significant family medical history presented with pre-syncope in the setting of hematemesis and melena. Her hemoglobin level dropped to 6.5 g/dL, prompting the administration of intravenous pantoprazole and resuscitation measures, which included multiple units of packed red blood cells. A computed tomography scan (CT) of the abdomen showed a 3.3 cm mass along the lesser curve of the stomach (Figure 1).

**Figure 2:**
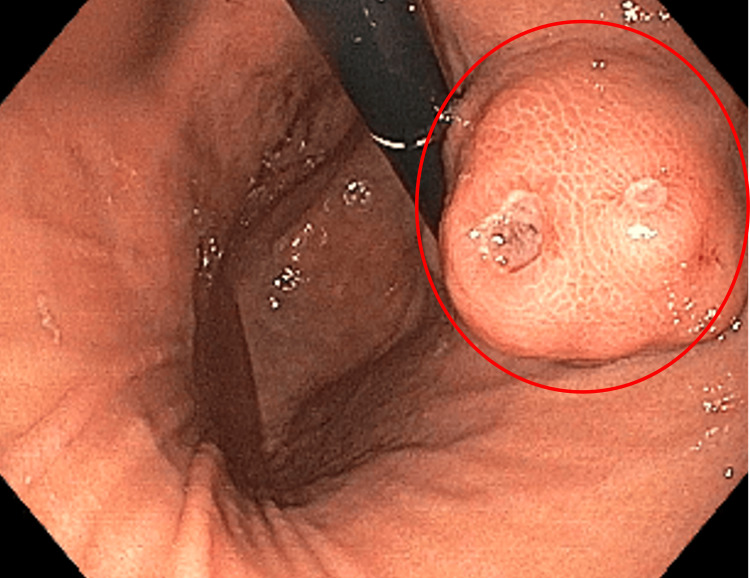
Ulcerated 3 cm GIST viewed in retroflexion GIST: gastrointestinal stromal tumor

 An esophagogastroduodenoscopy (EGD) showed an actively oozing, ulcerated, subepithelial mass near the GEJ (Figure 2).

**Figure 3:**
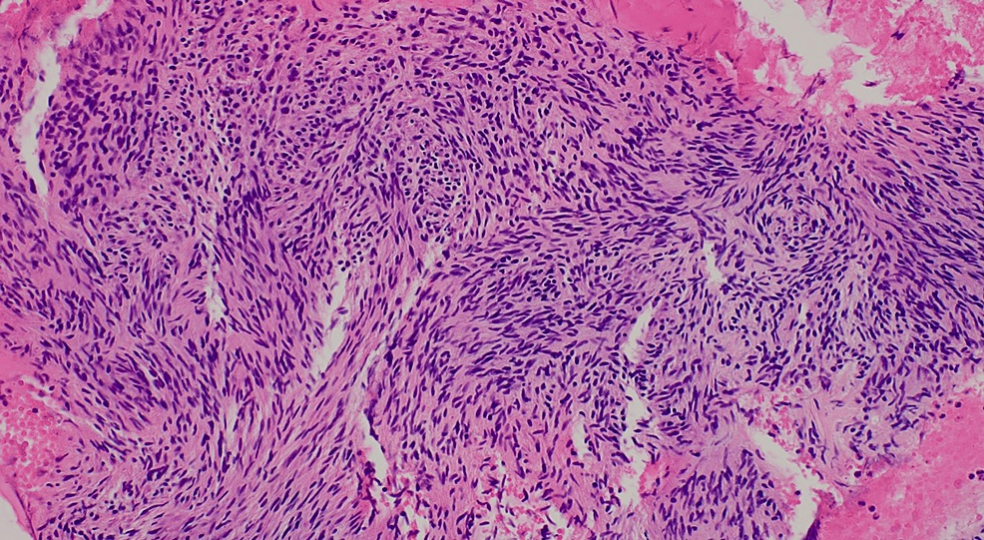
Spindle cells on cytology consistent with GIST GIST: gastrointestinal stromal tumor

The oozing was temporized with hemostatic powder spray, TC-325 (Hemospray, Cook Medical, Winston-Salem, NC). After a surgical oncology consultation, the patient underwent endoscopic ultrasound-guided-fine-needle aspiration of the mass. The cytology showed spindle cells with positive immunohistochemical staining for KIT and DOG-1, consistent with a GIST (Figure 3).

**Figure 4:**
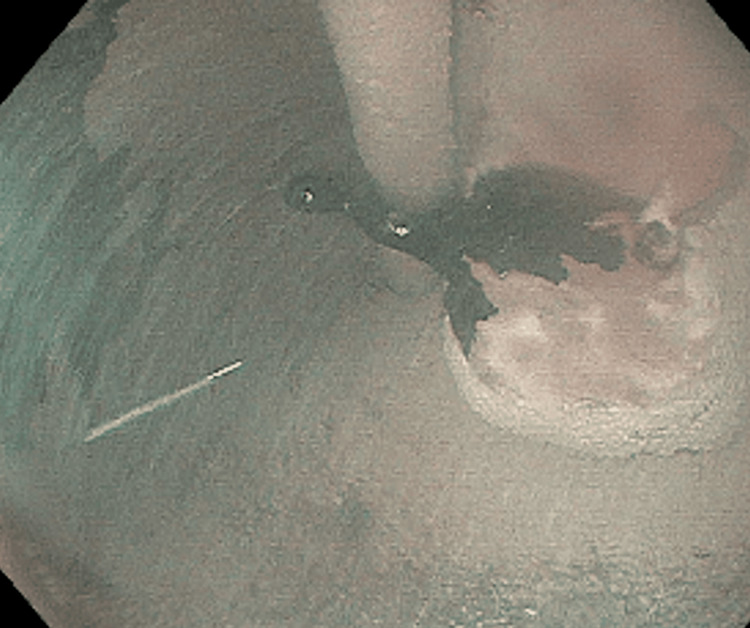
Hemostatic powder spray application to the bleeding GIST viewed in retroflexion GIST: gastrointestinal stromal tumor

The mitotic rate was 1 per 20 high-power fields. During the hospitalization, melena and an ongoing transfusion requirement prompted repeat EGDs utilizing the Olympus GIF-HQ190 scope (Olympus America Inc., Shinjuku City, Tokyo, Japan). Continued oozing was noted from the lesion and attempts to achieve hemostasis with endoscopic clips (including a 17 mm clip) were unsuccessful. Hemostasis was achieved by coating the lesion with TC-325 hemostatic powder spray (Figure 4). Administration of hemostatic powder spray near the GEJ with the endoscope in retroflexion caused it to adhere to the gastric mucosa. In the hands of an experienced endoscopist, the scope was safely removed, but it required a significant amount of force to pull the scope out while gently applying torquing motion. The patient’s gastrointestinal bleeding resolved, and she ceased to have a transfusion requirement. She was then started on imatinib mesylate 600 mg per day, with the plan to continue for six months prior to reevaluation for surgical resection.

The patient continued imatinib mesylate for six months, during which time she moved to another state. When followed up by telephone, her reported side effects were mild diarrhea, self-resolving lower extremity swelling, transient fatigue, and decreased appetite. Per reports, a follow-up CT scan at an outside hospital showed a significant reduction in the size of the tumor, and she subsequently underwent a laparoscopic partial gastrectomy. The final specimen revealed a 2.9 cm GIST with clear margins but with exon 11 and C-kit mutations. The mitotic rate was only 1 per high power field. Her recurrence risk was calculated to be 1.9%, and imatinib mesylate was discontinued. She is currently one year post-surgical resection and continues to do well. She is followed closely by her medical oncologist who is surveilling her with semi-annual CT abdomen/pelvis imaging to evaluate for any recurrence.

## Discussion

GISTs are very vascular tumors, and significant bleeding may require acute stabilization prior to further workup and definitive management. The location of the GIST can make endoscopic treatment of bleeding difficult using traditional modalities [10]. Hemostatic powder spray offers a good option to temporize the bleeding from GISTs. It has the benefit of covering a large surface area, can target difficult-to-reach places, and requires less technical skill than standard therapies.

This case adds to the growing body of literature which highlights the dangers of an EGD scope becoming stuck when hemostatic powder spray is deployed in a retroflexed manner. We postulate that the application of the hemostatic agent resulted in portions of the scope becoming adherent to the exposed gastric mucosa, thereby making the withdrawal of the EGD scope difficult. As the implementation of hemostatic powder spray becomes more widespread, gastroenterologists should be aware of this potential issue. We are unclear at this time if there was any true potential to cause esophageal/gastric tissue damage from the amount of force that was required to pull out the scope.

Following acute stabilization, workup and management should be based on the National Comprehensive Cancer Network (NCCN) guidelines for GISTs, as outlined in the following summary [2]. CT with intravenous contrast of the abdomen and pelvis is needed for initial localization and staging. Nodules with high-risk features (irregular border, cystic spaces, ulceration, echogenic foci, and heterogeneity) require complete surgical resection. Surveillance of nodules less than 2 cm and without high-risk features is acceptable and can be done by endoscopic ultrasound-guided fine-needle aspiration or core biopsy.

For nodules of 2 cm or greater, the goal is resection, and the decision of whether to utilize neoadjuvant chemotherapy depends on anticipated morbidity associated with surgery [5]. In our patient, the location and size of the GIST would have required a total gastrectomy to be curative, which carries a lot of morbidity which led to imatinib mesylate being started to reduce the size of the tumor. Bleeding GISTs are also felt to have a higher recurrence rate during the first five years after resection [6].

Approximately 80% of GISTs are associated with KIT receptor tyrosine kinase mutations and 5-10% with PDGFRA receptor tyrosine kinase mutations. Except for tumors with PDGFRA exon 18 mutations, most GISTs with KIT or PDGFRA mutations respond well to imatinib mesylate. Typically, the only contraindication to imatinib mesylate is an allergy to the medication [4]. There are case reports where therapy with imatinib mesylate itself has resulted in bleeding from the GIST. However, we propose that the sustained hemostasis in our patient was secondary to the imatinib mesylate therapy. She had been treated with the hemostatic powder spray previously, which only resulted in a temporary respite from her bleeding. It was only after the imatinib mesylate was started that her bleeding did not recur. Imatinib mesylate likely resulted in the tumor decreasing in size, which helped it achieve hemostasis.

## Conclusions

More research is needed on the potential hemostatic benefit of imatinib mesylate in bleeding GISTs, but this therapy should be a consideration for bleeding that is refractory to traditional therapies. In addition, hemostatic powder spray is a useful hemostatic modality that can be used after traditional hemostatic methods have failed. However, the potential adherence of the endoscope in the retroflexed position and limited visibility with hemostatic powder spray may limit its clinical use. 
